# Decreased protein binding of moxifloxacin in patients with sepsis?

**DOI:** 10.3205/id000029

**Published:** 2017-02-03

**Authors:** Christoph Dorn, Hartmuth Nowak, Caroline Weidemann, Stefan Martini, Markus Zeitlinger, Michael Adamzik, Frieder Kees

**Affiliations:** 1 Dept. of Clinical Pharmacy, Institute of Pharmacy, University of Regensburg, Germany; 2 Klinik für Anästhesiologie, Intensivmedizin und Schmerztherapie, Universitätsklinikum Knappschaftskrankenhaus Bochum, Germany; 3 Dept. of Clinical Pharmacology, Medical University of Vienna, Austria; 4 Dept. of Pharmacology, University of Regensburg, Germany

**Keywords:** fluoroquinolone, unbound fraction, ultrafiltration, septic shock

## Abstract

The mean (SD) unbound fraction of moxifloxacin in plasma from patients with severe sepsis or septic shock was determined by ultrafiltration to 85.5±3.0% (range 81.9 and 91.6%) indicating a decreased protein binding of moxifloxacin in this population compared with the value of 58–60% provided in the Summary of Product Characteristics. However, previous investigations neglected the influence of pH and temperature on the protein binding of moxifloxacin. Maintaining physiological conditions (pH 7.4, 37°C) – as in the present study – the unbound fraction of moxifloxacin in plasma from healthy volunteers was 84%. In contrast, the unbound fraction of moxifloxacin was 77% at 4°C and 66–68% in unbuffered plasma or at pH 8.5 in fair agreement with previously published data. PK/PD parameters e.g. *f*AUC/MIC or ratios between interstitial fluid and free plasma concentrations, which were obtained assuming a protein binding rate of moxifloxacin of 40% or more, should be revised.

## Introduction

Moxifloxacin is a fluoroquinolone with excellent activity against a broad spectrum of microorganisms involved in soft tissue infections [1]. Total tissue concentrations of moxifloxacin reach or exceed the corresponding plasma concentrations [2]. However, concentrations determined in tissue homogenate should be interpreted with caution, most importantly because no distinction can be made between the intra- and extracellular concentrations [3], [4]. Particularly, high concentrations of fluoroquinolones in tissue homogenate could be due to enrichment within the cells, whereas most bacterial infections occur in the extracellular space [4]. Therefore, measurement of interstitial concentrations, e.g. by microdialysis, is to be preferred whenever possible [5]. As the interstitial concentrations are basically free drug concentrations, the combination of measurement of the free plasma concentrations and the interstitial concentrations is the best way to get reliable results on the tissue concentrations of antibiotics and the tissue to plasma ratio. 

Previous studies have demonstrated that rapid or complete equilibration of antibiotics between plasma and tissue cannot be taken for granted for each clinical setting and patient population, particularly for those patients who suffer from substantially impaired local or systemic blood flow [6]. Hence, in the present study the kinetics of moxifloxacin were investigated in plasma as well as in interstitial fluid of patients with sepsis after single and multiple dosing using microdialysis technique. Here we report the results from the measurement of free moxifloxacin in plasma.

## Methods

### Patients and study design

The plasma samples were obtained from a single-center non-controlled pharmacokinetic study in adult ICU patients with severe sepsis or septic shock [7]. The aim of the study was to assess the plasma and tissue pharmacokinetics of moxifloxacin using microdialysis. The study was approved by the Ethics Committee of the Medical Faculty of the Ruhr-University Bochum (reference no. 4830-13). Written informed consent was obtained from either the patient or their legal representative. The patients received moxifloxacin 400 mg (Avalox 400 mg/250 mL solution for infusion, Bayer Vital GmbH, D-51368 Leverkusen, Germany) once daily as one-hour infusion. Arterial blood (LiHeparin monovette, Sarstedt Nümbrecht, Germany) and microdialysate was collected before infusion (baseline) and up to 10 hours on day 1, 3 and 5 of treatment. The blood was centrifuged and the plasma separated. All samples were stored at –80°C for up to 12 months, shipped to the analytical laboratory in dry ice and then stored further at –70°C for up to 2 months before analysis.

### Chemicals

Moxifloxacin hydrochloride (MXF-HCl) and Avalox 400 mg/250 mL (1 mL solution for infusion contains 1.6 mg/mL moxifloxacin) was obtained from Bayer Vital GmbH, Leverkusen. The internal standard gatifloxacin was obtained from Grünenthal GmbH, Aachen, Germany. Acetonitrile and methanol (both HPLC gradient grade) and the other chemicals were purchased from VWR, Darmstadt, Germany. HPLC grade water was produced with an Arium basic water purification system (Sartorius, Göttingen, Germany). Blank plasma was obtained from healthy volunteers.

### Stock solutions, calibrators (STD), and quality control samples

Moxifloxacin and gatifloxacin (IS) stock solutions (100 mg/L) were made in 0.01 M hydrochloric acid (HCl). Aliquots of the stock solutions were stored at –70°C and thawed before use. Residuals of thawed solutions were discarded. The standards (STD) and quality control (QC) samples were prepared by dilution in 0.1M HCl/MeOH 70:30 (v/v) and finally 1:20 (v/v) with plasma. STDs and QCs were stored at –70°C for no longer than 3 months until use. 

### Sample preparation

Total moxifloxacin was determined in all blood samples as published previously [8] with minor modifications. In brief, 100 µL internal standard (iS) solution (gatifloxacin 1 mg/L in 0.2% Na-EDTA, pH 6–7) was mixed with 100 µL plasma and 400 µL acetonitrile. The mixture was vortexed for 2–3 seconds, incubated for 15 min at 4°C and centrifuged. An aliquot (300 µL) of the supernatant was filled into the HPLC vials and diluted with 300 µL 0.01 M hydrochloric acid (in order to reduce peak broadening due to the high elution strength of the injection solution). An aliquot of 1–3 µL was injected into the HPLC system. Intra- and inter-assay imprecision as well as accuracy, based on in-process controls (2.5, 0.8 and 0.25 mg/L), were <4% and 100%, respectively. The concentrations of free moxifloxacin were determined by ultrafiltration applying a published method [9] in part of patient samples: prior to first administration (blank value), after 1 hour (end of infusion, high concentration) and after 10 hours (low concentration). In brief, 10 µL 3 M potassium phosphate buffer, pH 7.5, was pipetted into the ultrafiltration device (Nanosep Omega 10K, VWR, Darmstadt, Germany) and mixed with 300 µL plasma. Following incubation (10 min, 37°C, 100 g) in an Eppendorf 5417R centrifuge (Eppendorf, Hamburg, Germany) and centrifugation (20 min, 37°C, 1000 g), an aliquot (1–3 µL) of the ultrafiltrate (70–90 µL) was injected into the HPLC system. Unspecific adsorption of moxifloxacin to the UF device was assessed by filtration of 400 µL moxifloxacin 10, 3, 1, 0.3, 0.1, 0.03 or 0.01 mg/L in 0.1 M potassium phosphate buffer, pH 7.4 (2 min, 1000 g, ambient temperature). The recovery of moxifloxacin was constant over the whole range and amounted to 96.8±5.1% (mean±SD of 3 dilution series with 7 concentrations each). The intra-assay precision was not determined, as preliminary results revealed an intra-assay imprecision of <1%. The unbound fraction of moxifloxacin in a control pool (spiked with moxifloxacin 4 or 0.4 mg/L) from healthy volunteers analysed with each run was 82.6±1.8% (n=5) which corresponds to an imprecision of 1.9%. The accuracy cannot be specified, as the true value of the unbound fraction of the drug in plasma is not known. The potential concentration dependence of the protein binding of moxifloxacin was examined/checked analysing 6 independent dilution series in pooled plasma from healthy volunteers with moxifloxacin 10, 3, 1, 0.3, 0.1, 0.03 and 0.01 mg/L. 

### Chromatography

Chromatography was performed on a Prominence Modular HPLC equipped with a fluorimetric detector RF10AXL, set to ex/em 296/504 nm (Shimadzu, Duisburg, Germany). The autosampler was cooled to 6°C, the column temperature was 40°C. The separation system included a guard column 4x3 mm filled with Nucleoshell RP 18, 2.7 µ silicagel and an analytical column Nucleodur C18 HTec 3 µ, 125x4 mm. The mobile phase consisted of 800 ml water, 200 ml acetonitrile, 1600 mg tetrabutylammonium hydrogen sulfate, 800 µl phosphoric acid 85%, pH 3.0 with sodium hydroxide. Gatifloxacin (iS) eluted after 1.8 min and moxifloxacin after 2.4 min at a flow rate of 0.8 mL/min.

### Statistical analysis

GraphPad Prism 6.0 for MacOSX (GraphPad Software, La Jolla, CA, USA) was used for calculating nonparametric or parametric descriptive statistics. Least square analysis of dilution series was performed using the weighting factor 1/y^2^. Data are presented as mean (±SD), median or (interquartile) range, as appropriate. Unbound fraction (*f*u) in pool plasma has been calculated as measured free concentration/nominal concentration, whereas *f*u in patient plasma has been calculated as measured free concentration/measured total concentration in the respective plasma sample. 

## Results

**Figure 1:**
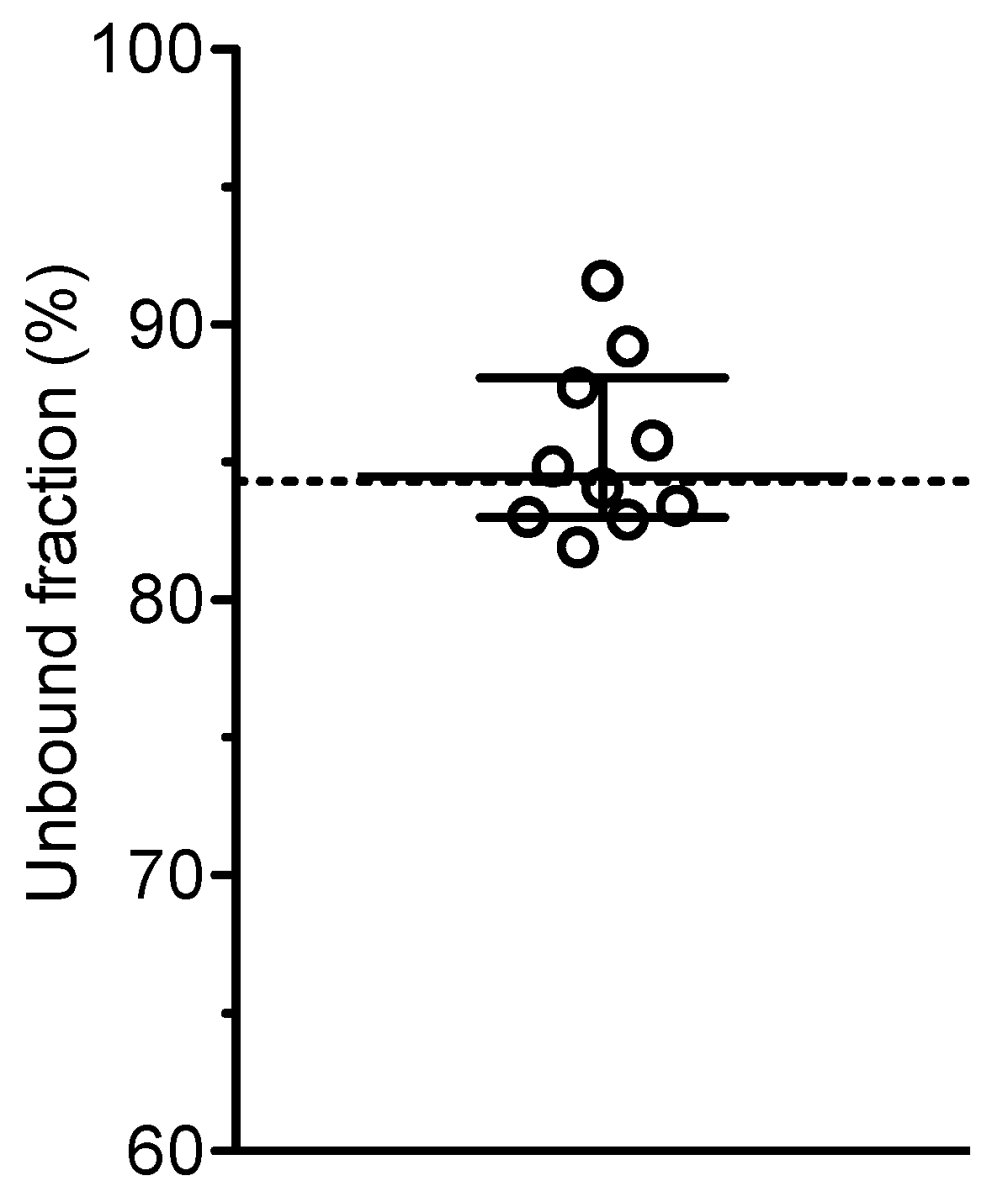
Unbound fraction (*f*u) of moxifloxacin in plasma of ICU patients with sepsis treated with moxifloxacin 400 mg i.v. once daily Median, IQR, circles = mean values of 6 samples per patient, dotted line = *f*u in pool plasma from healthy volunteers

Unspecific binding of moxifloxacin onto the ultrafiltration membrane was negligible. Therefore, the results are not corrected for unspecific binding. The protein binding of moxifloxacin in pool plasma from the healthy volunteers was independent of the concentration. The dilution series (range 10.0–0.01 mg/L) was perfectly linear (r=1.000, y=0.843*x+0.000) revealing a mean unbound fraction of 84.3%. Therefore, we considered it sufficient to measure the free plasma concentrations of moxifloxacin only in a part of the patient samples (after 1 hour and after 10 hours). Additionally, as the total plasma concentrations of moxifloxacin were similar on day 1, 3 and 5 (peak concentrations averaged over all 10 patients were 3.16±0.27 mg/L at the end of infusion (1 hour) and 1.01±0.13 mg/L after 10 hours, respectively), the concentrations of each patient were averaged to calculate the individual unbound fraction. Thus, the mean unbound fraction of moxifloxacin in plasma of the 10 patients was 85.5±3.0% (range 81.9 and 91.6%) with a mean intraindividual coefficient of variation of 2.4% (Figure 1 (Fig. 1)).

**Figure 2:**
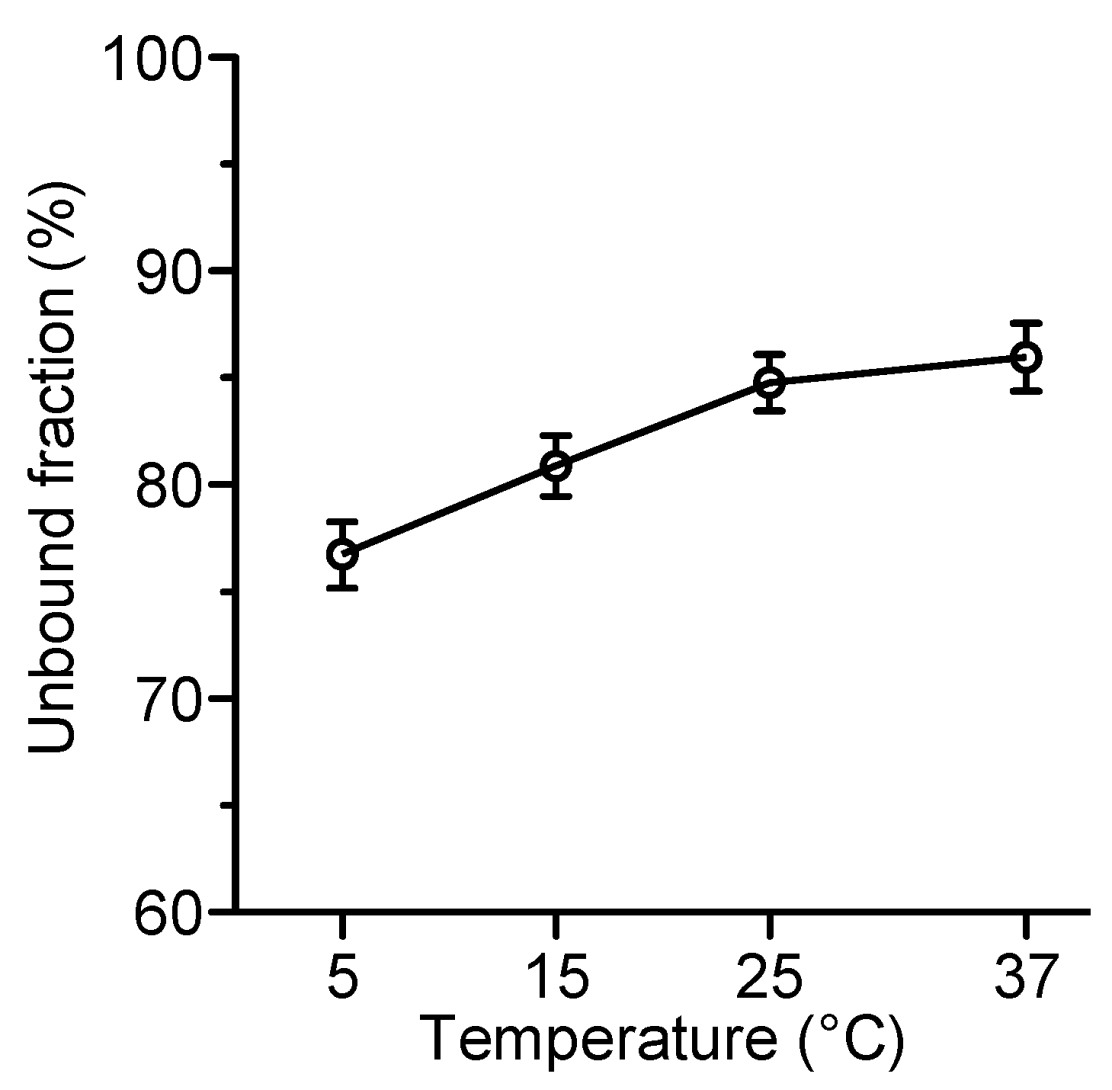
Influence of temperature on the protein binding of moxifloxacin in human plasma, buffered with potassium phosphate to 7.4, as determined by ultrafiltration. Mean±SD of 4 independent experiments with moxifloxacin 3 or 0.3 mg/L (n=8)

The unbound fraction was higher than the value of 58–60% provided by the manufacturer (Fachinformation Avalox 400 mg/250 ml Infusionslösung, date 06/2015, Bayer Vital, Leverkusen, Germany). In order to explain the discrepancy, the influence of experimental variables such as temperature and pH were investigated. Temperature had a slight influence on the unbound fraction of moxifloxacin which increased from 77% at 5°C to 85–86% at 25–37°C (Figure 2 (Fig. 2)).

**Figure 3:**
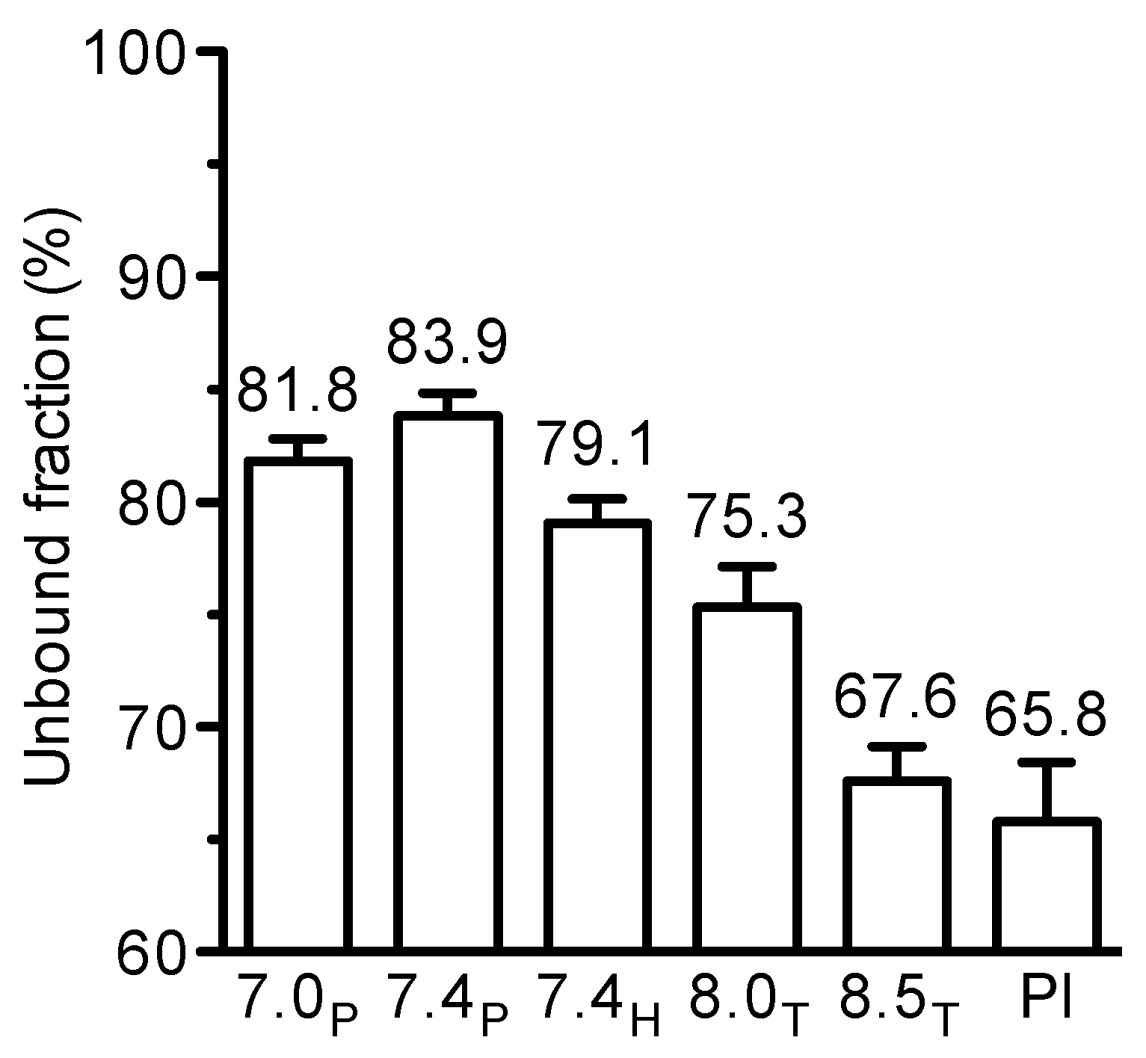
Influence of pH on the protein binding of moxifloxacin in human plasma (at 37°C) as determined by ultrafiltration. Mean±SD of 4 independent experiments with moxifloxacin 3 or 0.3 mg/L (total n=8) Abbr.: P phosphate, H HEPES, T TRIS, Pl plasma unbuffered

To examine the influence of pH, plasma was spiked with moxifloxacin 3 or 0.3 mg/L and buffered with potassium phosphate, HEPES or TRIS to pH ranging from 7.0 to 8.5 as described recently [10]. For comparison, unbuffered plasma was analysed as well. The unbound fraction was 79–84% at pH 7.0 to 7.4 and decreased to 66–68% at pH 8.5 or in unbuffered plasma (Figure 3 (Fig. 3)). 

## Discussion

Antiinfective drugs are commonly evaluated on the basis of pharmacokinetic/pharmacodynamic (PK/PD) indices, all of which are based on a comparison of its plasma concentrations and the minimal inhibitory concentration (MIC) against the pathogen. However, plasma protein binding affects the antimicrobial activity of fluoroquinolones [11]. Consequently, all PK/PD indices should refer to the unbound (non-protein bound) fraction of a drug [12]. In addition, the concentrations in the interstitial fluid are considered to equal the free plasma concentrations. Therefore, it was reasonable to determine not only the concentrations of moxifloxacin in the intersitial fluid in the present microdialysis study, but also the free plasma concentrations in a subset of plasma samples.

The plasma protein binding of moxifloxacin in man is about 40% according to the SPC and previously published data [11], [13], [14]. Our results indicate a lower plasma protein binding of moxifloxacin in patients with sepsis. Hypoalbuminaemia is often observed in critically ill patients [15], [16] and is readily used to explain reduced protein binding in such patients [16]. However, the protein binding of moxifloxacin in plasma of healthy volunteers was also about 15% only. On the other hand, the mean unbound fraction of moxifloxacin was lower in plasma buffered to pH 8.5 or in plain plasma, where the pH raises above 8 or reaches even 9 after preparation and storage [17]. This behaviour is typical for basic drugs which become more lipophilic at alkaline pH [17]. Accordingly, no pH dependency has been observed with neutral antimicrobials such as linezolid or fluconazole [10], whereas the protein binding of tigecycline, a tetracycline with an excess of amino functions, increases dramatically at pH>8 [18]. Fluoroquinolones are zwitterions and most lipophilic at the isoelectric point, which is 8.0 for moxifloxacin. This unusual high isoelectric point of moxifloxacin may explain the higher protein binding of moxifloxacin at elevated pH. Accordingly, also the penetration into the alkalotic human prostate secret was highest for moxifloxacin compared with other fluoroquinolones [19]. To sum up, strict pH and temperature control is necessary when studying protein binding [17]. There are numerous publications reporting and discussing data that have been produced without sufficient control of these parameters [17] including moxifloxacin [11], [20]. There is one publication reporting a protein binding of 54±14% in human serum despite adjusting the pH to 7.0–7.5 and pre-incubation of serum at 37°C [14]. However, hydrochloric acid has been used for pH adjustment, which has no buffer properties at physiological pH. Moreover, the high coefficient of variation (26%) indicates that the method used was imprecise and presumably not reliable. To sum up, it should be appropriate to assume an unbound fraction of moxifloxacin of 85% in man concerning PK/PD calculations [12].

We use phosphate buffer in our standard procedure [9] in analogy to the buffer system used in equilibrium dialysis [21], which still is considered as “gold standard” for the determination of free drug concentrations [22]. However, experiments with tigecycline revealed a significant higher unbound fraction, when the plasma was buffered with phosphate compared with HEPES [18]. We assigned this observation to competition for calcium between phosphate and tigecycline. It is well known that besides tetracyclines also fluoroquinolones form chelate complexes with multivalent metal ions [23], [24]. As a surprising consequence of the complex formation between tetracyclines or fluoroquinolones and calcium, lower plasma concentrations of tigecycline and ciprofloxacin were measured when blood was collected in EDTA-coated tubes instead of heparin-coated tubes. Obviously, EDTA binds plasma calcium. As a consequence, non-chelated tigecycline or ciprofloxacine diffuse into the blood cells thus lowering their plasma concentrations [25]. Moreover, as calcium binds to albumin in a pH dependent manner [26], interference of phosphate buffer with the protein binding of drugs such as tetracyclines or fluoroquinolones is conceivable. Concerning moxifloxacin, the difference of the unbound fraction in phosphate or HEPES buffered plasma was statistically significant but small (84 vs 79%) and clinically irrelevant. In addition, one has to take into account that the pH cannot be exactly maintained at 7.4 either with phosphate or with HEPES. There is a small drift (ca. 0.3 pH units) upwards during ultrafiltration for 20 min at 37°C with phosphate [9] due to evaporation of carbon dioxide. Furthermore, HEPES shows a negative pH drift with temperature in contrast to phosphate [27]. Therefore, the statistically significant difference may partly be explained by differences in the actual pH during ultrafiltration in phosphate and HEPES buffered plasma, respectively.

## Conclusion

The clinical relevance of the apparently higher unbound fraction of moxifloxacin as claimed in our study (85% compared with 60% as provided in the SPC) may be limited. However, as all PK/PD indices should be referenced to the unbound (non-protein bound) fraction of the drug [12], the conclusions from published studies using the 50–60% values would be slightly different. For example, the 90% probability of target attainment (*f*AUC/MIC=100 for Gram-negative pathogens) in patients with diabetic foot infections was achieved for pathogens with MIC≤0.125 [4]. Taking an unbound fraction of 85% as basis, a slightly more favourable MIC≤0.18 would result. Moreover, based on microdialysis experiments, the mean ratio of AUC_tissue_/*f*AUC_plasma_ in patients or healthy subjects was calculated between 0.7 and 1.2 [6], [28], [29] which would change to 0.45 and 0.75.

## Notes

### Acknowledgement

The study was supported by an unrestricted grant from Paul-Ehrlich-Gesellschaft für Chemotherapie.

### Competing interests

The authors declare that they have no competing interests.
